# Supplementary material for: Growth Curves and Body Condition of Young Cats and Their Relation to Maternal Body Condition
Source: Animals (Basel). 2022 May 27;12(11):1373. doi: 10.3390/ani12111373 (PMC9179872; doi:10.3390/ani12111373)
Supplement: Supplementary file 1 [file animals-12-01373-s001.zip › animals-1737827-supplementary.pdf]

Table S1. Mean live weight (g) and standard deviation (g) per month in relation to sex, kittens' BCS at 8 months and maternal phenotype.

	Group	N	BW	1	2	3	4	5	6	7	8	9	10	11	12
**Male**	OM	15	111 ± 16	527 ± 108	1065 ± 240	1881 ± 321	2826 ± 548	3586 ± 589	4328 ± 619	4903 ± 725	5258 ± 710	4978 ± 851	5117 ± 665	4947 ± 550	4793 ± 239
	VM	9	107 ± 14	504 ± 78	1040 ± 182	1878 ± 330	2701 ± 450	3419 ± 621	4044 ± 720	4618 ± 826	4907 ± 891	4330 ± 782	4450 ± 803	4253 ± 924	3987 ± 833
	LM	24	106 ± 11	504 ± 81	1072 ± 207	1914 ± 289	2628 ± 387	3413 ± 370	4009 ± 389	4442 ± 451	4831 ± 389	4801 ± 377	4764 ± 398	4760 ± 453	4643 ± 441
	O	18	110 ± 15	535 ± 92	1129 ± 187	2015 ± 279	2929 ± 429	3753 ± 454	4471 ± 505	5072 ± 601	5453 ± 554	4989 ± 592	4870 ± 528	4864 ± 423	4757 ± 268
	L	30	107 ± 12	497 ± 85	1025 ± 215	1826 ± 215	2567 ± 295	3298 ± 438	3902 ± 455	4347 ± 505	4675 ± 466	4667 ± 504	4719 ± 502	4639 ± 602	4486 ± 595
	Total	48	108 ± 13	511 ± 88	1064 ± 210	1897 ± 301	2703 ± 453	3468 ± 492	4115 ± 546	4619 ± 644	4972 ± 626	4770 ± 541	4770 ± 503	4714 ± 549	4567 ± 527
**Female**	OM	8	109 ± 11	522 ± 56	1076 ± 116	1791 ± 262	2533 ± 334	3065 ± 352	3482 ± 480	3591 ± 614	3719 ± 616	3670 ± 694	3675 ± 468	3613 ± 536	3653 ± 575
	VM	7	90 ± 9	488 ± 60	1011 ± 140	1632 ± 218	2128 ± 122	2564 ± 136	2870 ± 139	3150 ± 180	3257 ± 273	3405 ± 217	3458 ± 201	3178 ± 172	2930 ± 42
	LM	22	103 ± 15	455 ± 88	959 ± 262	1689 ± 224	2271 ± 256	2674 ± 266	2890 ± 266	3071 ± 364	3154 ± 419	3383 ± 475	3459 ± 475	3492 ± 621	3660 ± 645
	O	11	98 ± 16	495 ± 90	1043 ± 132	1792 ± 256	2348 ± 347	2917 ± 342	3276 ± 426	3581 ± 412	3662 ± 463	3778 ± 497	3680 ± 415	3737 ± 597	3683 ± 678
	L	26	103 ± 13	468 ± 77	974 ± 247	1660 ± 213	2281 ± 258	2662 ± 279	2903 ± 319	3036 ± 360	3138 ± 410	3274 ± 402	3413 ± 488	3335 ± 508	3455 ± 614
	Total	37	102 ± 14	476 ± 81	994 ± 220	1700 ± 231	2301 ± 284	2738 ± 317	3014 ± 389	3198 ± 448	3284 ± 481	3449 ± 492	3498 ± 474	3450 ± 551	3520 ± 624

O = kitten BCS ≥ 6 at 8 months, L = kitten BCS < 6 at 8 months, OM = maternal BCS always ≥ 6, VM = maternal BCS differing between < and ≥ 6, LM = maternal BCS always < 6, BW: birthweight

Table S2. Mean daily weight gain (g) and standard deviation (g) per month, calculated from total weights per month, in relation to sex, kittens' BCS at 8 months and maternal phenotype.

	Group	N	0 - 1	1 - 2	2 - 3	3 - 4	4 - 5	5 - 6	6 - 7	7 - 8	8 - 9	9 - 10	10 - 11	11 - 12
**Male**	OM	15	14 ± 4	17 ± 5	27 ± 7	31 ± 10	25 ± 7	25 ± 7	19 ± 7	8 ± 8	1 ± 4	-3 ± 7	-6 ± 5	-5 ± 10
	VM	9	13 ± 3	17 ± 7	28 ± 7	27 ± 5	23 ± 6	21 ± 5	19 ± 5	10 ± 4	-1 ± 10	4 ± 2	-7 ± 7	-9 ± 4
	LM	24	13 ± 3	18 ± 5	27 ± 9	23 ± 8	26 ± 6	20 ± 8	14 ± 6	13 ± 6	2 ± 13	-1 ± 5	0 ± 6	-4 ± 6
	O	18	14 ± 3	19 ± 5	29 ± 7	30 ± 7	27 ± 5	24 ± 7	19 ± 6	12 ± 7	-8 ± 12	-4 ± 7	0 ± 6	-4 ± 8
	L	30	13 ± 3	17 ± 6	26 ± 8	24 ± 9	24 ± 7	20 ± 7	14 ± 6	10 ± 6	7 ± 7	1 ± 3	-3 ± 5	-5 ± 6
	Total	48	13 ± 3	18 ± 5	27 ± 8	26 ± 9	25 ± 6	22 ± 7	16 ± 6	11 ± 7	2 ± 11	-1 ± 5	-2 ± 6	-5 ± 7
**Female**	OM	8	13 ± 2	18 ± 4	23 ± 6	24 ± 6	17 ± 7	14 ± 9	3 ± 5	6 ± 7	4 ± 5	8 ± 7	-4 ± 7	1 ± 2
	VM	7	13 ± 2	17 ± 4	20 ± 4	16 ± 5	14 ± 3	10 ± 3	9 ± 3	3 ± 4	1 ± 6	2 ± 3	-10 ± 6	-8 ± 4
	LM	22	12 ± 3	16 ± 7	26 ± 7	19 ± 6	13 ± 3	7 ± 7	6 ± 7	3 ± 5	5 ± 6	2 ± 9	1 ± 6	5 ± 11
	O	11	13 ± 3	18 ± 3	24 ± 5	18 ± 6	18 ± 4	12 ± 11	10 ± 8	4 ± 4	2 ± 4	1 ± 5	1 ± 10	-2 ± 8
	L	26	12 ± 2	16 ± 7	25 ± 7	20 ± 6	13 ± 3	8 ± 4	4 ± 4	3 ± 6	5 ± 6	4 ± 8	-3 ± 6	4 ± 11
	Total	37	12 ± 2	17 ± 6	24 ± 7	20 ± 6	14 ± 4	9 ± 7	6 ± 6	4 ± 5	4 ± 6	3 ± 8	-2 ± 7	2 ± 10

O = kitten BCS ≥ 6 at 8 months, L = kitten BCS < 6 at 8 months, OM = maternal BCS always ≥ 6, VM = maternal BCS differing between < and ≥ 6, LM = maternal BCS always < 6, BW: birthweight
